# Emergency retroperitoneal laparoscopic partial nephrectomy for ruptured renal angiomyolipomas: a retrospective single-center series of 15 cases

**DOI:** 10.1186/s12893-020-00723-w

**Published:** 2020-03-30

**Authors:** Wei He, Xiaoxu Chen, Haiyong Ji, Jianwei Wang, Zhihong Niu

**Affiliations:** 1 Department of Urology, Shandong Provincial Hospital Affiliated to Shandong First Medical University, 324 Jingwu Road, Huaiyin District, Jinan, 250021 Shandong province China; 2 Department of Urology, Shandong Provincial Hospital Affiliated to Shandong University, Jinan, Shandong province China; 3 The Third Department of Surgery, Ningjin People’s Hospital, Ningjin County, Dezhou, Shandong province China; 4 Department of Urology, Shandong Provincial ENT Hospital Affiliated to Shandong University, Jinan, Shandong province China

## Abstract

**Background:**

To assess the safety, tumor control and renal function preservation of the emergency retroperitoneal laparoscopic partial nephrectomy (LPN) for ruptured renal angiomyolipoma (AML) and summarize our single-center initial experience.

**Methods:**

We performed a retrospective analysis of 15 patients pathologically confirmed renal AML treated with emergency retroperitoneal LPN between January 2016 and May 2019. The patient demographics, operation time, blood loss, transfusion requirements, complications and other surgical parameters were analyzed. Follow-up was performed by serum creatinine and imaging modalities.

**Results:**

Fifteen patients were performed with emergency LPN with the median age 41.6 years. The mean size of the renal AMLs was 7.8 cm. The mean size of the retroperitoneal hematomas was 8.5 cm. All the emergency surgeries were performed successfully without any conversion to nephrectomy or open surgery. The mean operative time was 101 min. The mean warm ischemia time was 28 min. The mean estimated blood loss was 311 ml. Five patients required intraoperative blood transfusion (33.3%, 5/15). The mean transfused RBC was 4 U (range 2-6 U), and the mean transfused plasma was 200 ml (range 200-400 ml). The mean drainage duration was 3 days (range 2–5 days). The mean postoperative hospitalization was 4.7 days. No patients experienced intraoperative complications. The mean serum creatine was slightly higher after surgery (53.1 vs. 55.9 μmol/L). One patient had postoperative perirenal fluid collection. No patients needed dialysis. No recurrence was observed in the patients at the median follow-up of 24.1 months.

**Conclusions:**

Our initial experience shows that the emergency retroperitoneal LPN is a safe, minimally invasive procedure for emergency patients with ruptured renal AMLs. It could be considered as an effective alternative to renal artery embolization in selected emergency patients.

## Background

Renal angiomyolipoma (AML) is a common benign mesenchymal tumor consisting of variable proportions of three elements: peculiar blood vessels, smooth muscle fibers and adipocyte clusters; the incidence is 0.4% in the general population, accounting for ~ 3% of renal masses [1]. Renal AMLs are more prevalent in women than in men. Approximately, about 80% of renal AMLs are sporadic, and the remaining 20% of cases are diagnosed as tuberous sclerosis complex (TSC), which is an autosomal dominant disorder characterized by a series of benign tumors involving multiple systems [2]. The blood vessels in the tumors lack the internal elastic lamina, and the smooth muscle is disrupted, leading the renal AMLs to be prone to aneurysm formation and rupture [3]. Most renal AMLs are asymptomatic and diagnosed as incidentalomas during abdominal ultrasonography or computed tomography scans. The most severe complication related to renal AMLs is life-threatening retroperitoneal hemorrhage. Prophylactic or therapeutic treatment strategies are available spanning the spectrum of conservative, embolization or surgery based on the presence of symptoms and the lesion size.

Renal artery embolization is favored as the first-line therapy for bleeding renal AMLs and is used as a prophylactic intervention for women of childbearing age and for large renal AMLs before surgery [2]. Surgical intervention is commonly utilized as a second-line treatment for the renal AMLs with symptoms of any severity, suspicion of malignancy, and larger tumors. This method is reserved for patients with refractory symptoms, failure of embolization, and as complex renal artery anatomy unsuitable for embolization [4]. Based on the current opinion [5], it is proposed that the emergency surgical management would likely result in nephrectomy; until now, no study has focused on minimal invasive surgery for renal AMLs in emergency settings.

As is the case in the artificial pneumoperitoneum, the hyperbaric effect can decrease the oozing from the ruptured tumor, making it possible for immediate surgical treatment. Based on this premise, we employed emergency retroperitoneal laparoscopic partial nephrectomy (LPN) in the management of bleeding renal AMLs. Here, we report 15 cases of retroperitoneal hemorrhage caused by ruptured renal AMLs treated with emergency LPN in our center. This study is the first series in the literature focusing on emergency LPN management for bleeding renal AMLs, with special attention paid to feasibility, efficacy, safety, tumor control and renal function.

## Methods

### Patient population

**Fig. 1:**
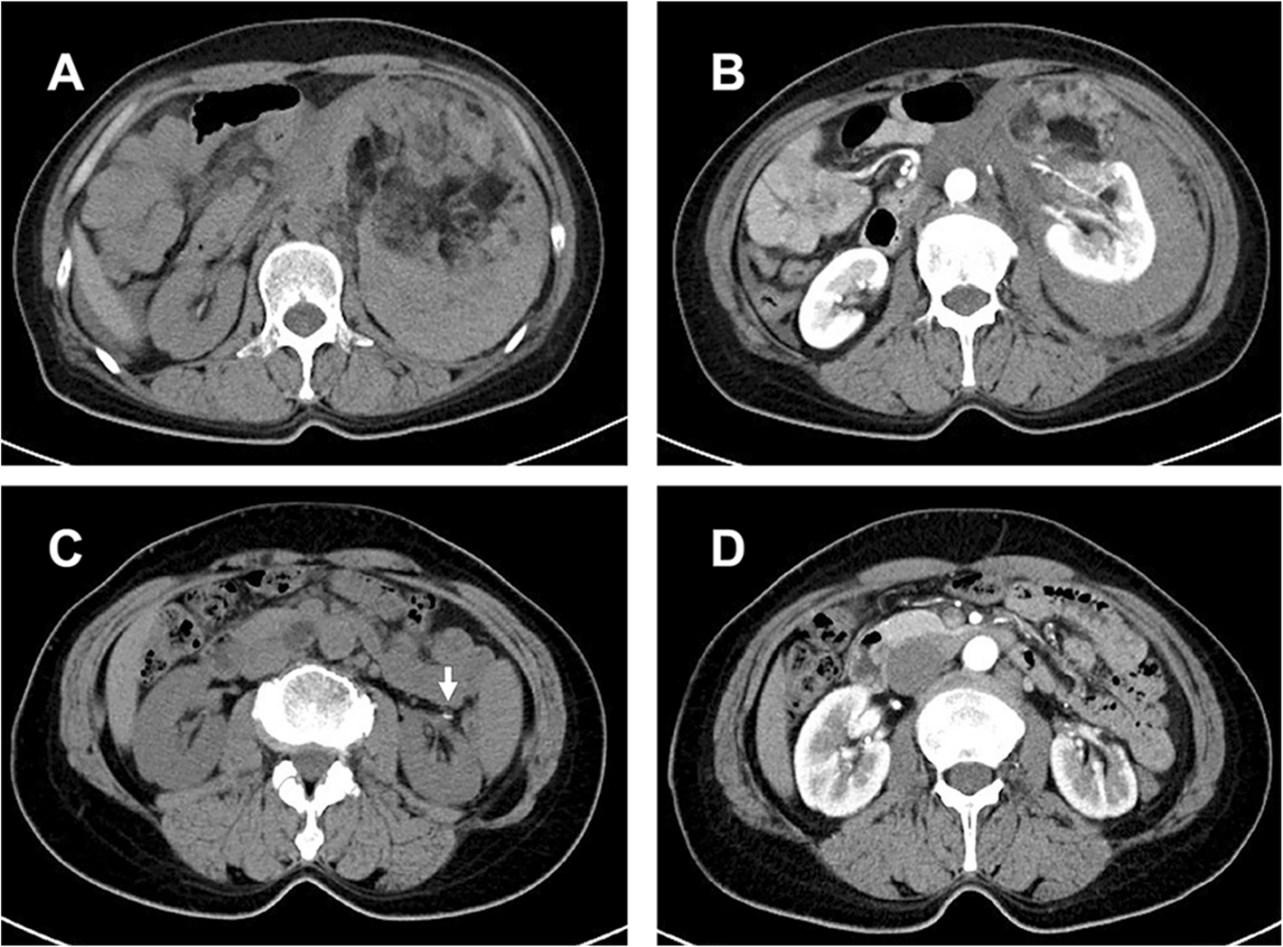
A ruptured left renal AML. The preoperative CT scans (**a** and **b**) show the abnormal morphology of the left kidney with a mixture of adipose tissue, ruptured cortex and retroperitoneal hematoma. The postoperative CT scans (**c** and **d**) (1 year after surgery) show left renal cortex defect and the hem-o-loc clip (white arrow). The ruptured renal AML was resected completely, and no recurrence was found

The study was approved by the institutional review board of Shandong Provincial Hospital (No. 2019–151). We retrospectively reviewed our institutional registry to identify patients treated with emergency LPN for ruptured renal AMLs over the period of January 2016 to May 2019. All the participants were informed and freely made decisions on the involvement by giving them sufficient information about the treatment and alternative treatments for their choosing. Written consent was obtained at the time of treatment. The methods were carried out in accordance with the approved guidelines. In this retrospective study, patients were admitted to our emergency department due to sudden flank pain with various degrees of shock symptoms and then were confirmed to have retroperitoneal hemorrhage caused by the rupture of renal AMLs. After receiving effective fluids, blood transfusion resuscitation, and perioperative preparation, the patients defined as hemodynamically stable received urgent surgical intervention without preoperative renal artery embolization. All the patients were preoperatively assessed in terms of the artery anatomy, tumor characteristics, size and location with either CT or MRI (Fig. 1a and b).

### Surgical technique

**Fig. 2:**
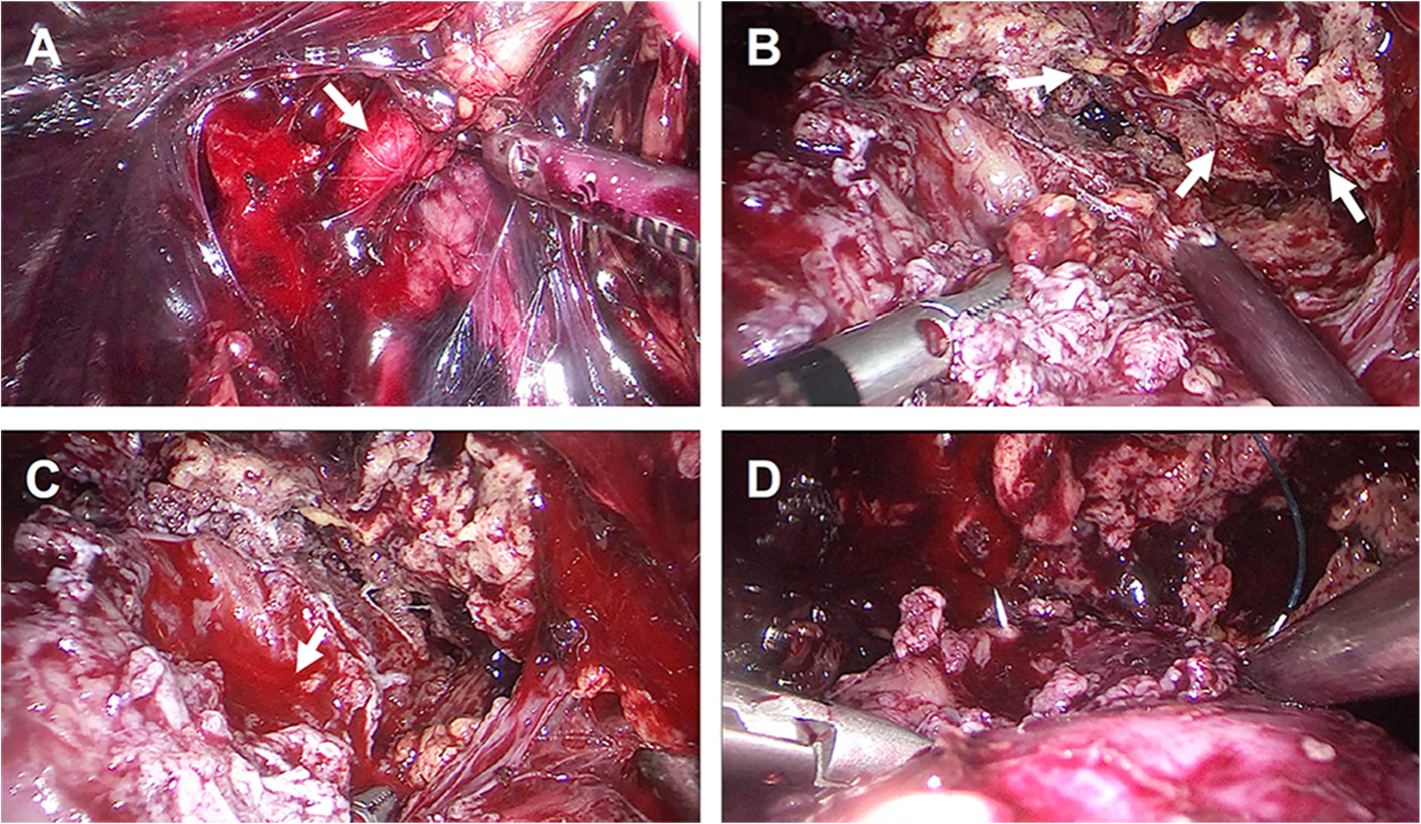
The intraoperative screenshot of the urgent laparoscopic nephron-sparing surgery. **a** The renal artery (white arrow) was dissected and exposed along the psoas. **b** and **c** After blocking the renal artery, excision and aspiration of the ruptured tumor (white arrow in **b** and hematoma were performed for adequate exposure of the tumor bed (white arrow in **c**). **d** Renorrhaphy and then complete resection the tumor and perirenal hematoma were performed

All the surgeries were performed by a single veteran urologist (Dr. Zhihong Niu, > 700 laparoscopic partial nephrectomies) using a retroperitoneal laparoscopic technique. The patients were positioned in the lateral decubitus position with overextension. The retroperitoneal space was created using the index finger and a glove-made balloon. A typical retroperitoneal laparoscopic 3-trocar technique was utilized [6]. The renal artery priority strategy was adopted. First, the renal artery was dissected and exposed along the psoas (Fig. 2a). The renal vein was left intact. Next, the ruptured tumor tissue and perinephric blood clot around the tumor bed were aspirated, and the perinephric tissue was dissected to adequately expose the margin of the tumor (Fig. 2b). Extensive dissection of the tumor and blood clot was avoided before renorrhaphy because extensive mobilization without renal artery blocking might increase bleeding. In addition, once the renal artery is clamped, the extensive mobilization of the tumor might prolong the renal ischemic time. After adequate exposure of the root, the renal artery was temporarily blocked by bulldog clamping without renal vein blockage. We resected the bottom of the tumor from the kidney. The dissected perirenal tumor and hematoma were “pushed away” from the kidney by the artificial pneumoperitoneum, making more space for renorrhaphy (Fig. 2c). Subsequently, the cut parenchymal surface was approximated with a 3–0 V-loc™ barbed suture (Medtronic, Minneapolis, MN). The renal capsule was reconstructed using 2–0 barbed sutures (Fig. 2d). After the kidney blood supply was restored and hemostasis was achieved, we completely detached the perinephric angiolipoma and retroperitoneal hematoma from the perinephric fat. The specimen was collected, removed with a retrieval bag, and sent for intraoperative frozen-section diagnosis. Retroperitoneal drainage was placed near the surgical field.

The follow-up consisted of laboratory findings, regular ultrasonography and CT scans (Fig. 1c and d). The follow-up protocol was scheduled 3 and 6 months postoperatively and annually thereafter. Patient demographics, clinical features, estimated blood loss, transfusion requirements and other perioperative data were recorded and analyzed.

## Results

The patient demographics and outcomes were summarized in Table 1 (detailed data in Supplementary Table 1). Fifteen patients with a median age 41.6 years underwent emergency LPN. Thirteen (86.7%, 13/15) patients were women, and two (13.3%, 2/15) patients were men. Six (40%, 6/15) patients underwent right-sided LPN, and nine (60%) underwent left-sided LPN. One patient (6.7%, 1/15) was diagnosed with TSC. The mean size of the renal AML was 7.8 cm. The mean size of the retroperitoneal hematoma was 8.5 cm. All the urgent LPNs were performed successfully without any conversion to nephrectomy or open surgery. The mean operative time was 101 min. The mean warm ischemia time (WIT) was 28 min. The mean amount of estimated blood loss was 311 ml. Five patients required intraoperative blood transfusions (33.3%, 5/15). The mean transfused RBC count was 4 U (range 2–6 U), and the mean transfused plasma volume was 200 ml (range 200–400 ml). The mean drainage duration was 3 days (range 2–5 days). The mean length of postoperative hospitalization was 4.7 days. No patients experienced intraoperative complications such as vessel injury, pneumothorax, bleeding or hematoma in this series. The frozen-section analyses performed for all the patients demonstrated renal AMLs. The mean serum creatine level was slightly higher after surgery (53.1 vs. 55.9 μmol/L). No patients needed dialysis. There was one patient with a postoperative complication in the form of perirenal fluid collection who recovered with conservative treatment. The median follow-up time was 24.1 months. During the follow-up, no recurrence was observed in the patients. The postoperative histologic pathology confirmed renal AML in all patients.

**Table 1:** Patient demographics

Patients (n)	15
Age (years)	41.6 ± 10.3
Gender	
Male/female	2 (13.3%) / 13 (86.7%)
BMI Kg/M^2^	23.4(19.4–33.0)
Tuberous Sclerosis	1 (6.7%)
Side of surgery	
Right/Left	6 (40%) / 9 (60%)
Size of tumor (cm)	7.8 ± 2.8
Size of hematoma (cm)	8.5 ± 3.5
Preoperative SCr (umol/L)	53.1 ± 7.7
Preoperative HB (g/L)	109.3 ± 12
Operative time (minute)	101 ± 15
WIT (minute)	28 ± 5
EBL (ml)	311 ± 217
Blood transfusion	5/15 (33.3%)
Introperative RBC transfusion (U)	4 (2–6)
Introperative Plasma transfusion (ml)	200 (200–400)
Postoperative SCr (umol/L)	55.9 ± 7.3
Postoperative HB (g/L)	98.3 ± 9.4
Drainage days	3 (2–5)
Postoperative Hospitalization	4.7 ± 1.1
Mean follow-up (month)	24.1 ± 13.2
Complications	
Perirenal fluid	1
Recurrence	0

*SCr* serum creatine, *HB* hemoglobin, *WIT* warm ischemic time, *EBL* estimated blood loss

## Discussion

Although a small subset of angiomyolipoma may behave more aggressively, AML is the most frequent benign neoplasm of the kidney. Renal AML has a female predominance, as the male to female ratio is 1:3–1:4 [7]. Approximately 80% of AMLs are identified as isolated entities, which tend to be single, small and rarely cause significant morbidity [8]. In contrast, the remaining 20% are associated with TSC, a hereditary tumor that affects both sexes equally and tends to be large, multiple, and bilateral, likely to be prone to hemorrhage and more aggressive [9]. TSC is an autosomal dominant disease characterized by benign tumors in multiple organs, including the brain, skin, lungs and kidneys [10]. Most renal AMLs are asymptomatic and are incidentally diagnosed, owing to the increased use of imaging modalities. The most straightforward diagnosis of renal AMLs depends on identifying a fat component on plain CT scans or a high signal on unenhanced MRI T1-weighted images. However, a minority of renal AMLs lack detectable adipose tissue, making them difficult to distinguish from renal cancer.

The clinical manifestations of renal AMLs are related to the tumor size, and most patients with small lesions have no clinical symptoms. When the size is larger than 4 cm, the tumors are prone to rupture spontaneously [11]. The classic triad associated with renal AMLs includes flank pain, a palpable mass and gross hematuria; other clinical presentations include vomiting, nausea, fever, hypertension and anemia. The most severe complication of renal AML is retroperitoneal hemorrhage presenting with sudden-onset flank pain, and one-third of patients may develop hypovolemic shock [7]. Such situations constitute a medical emergency, and the urgent management is indicated.

The treatments for bleeding AMLs include conservative treatment, renal artery embolization and immediate or delayed surgery after embolization [12]. Selective renal artery embolization is favored in patients, especially in the event of acute bleeding and hemodynamic instability in emergency conditions [1, 9]. It is proposed that the embolization possesses several advantages, including minimal invasiveness, low complication rates, good renal function preservation, and surgery avoidance.

Although, we agree that arterial embolization is useful for the control of bleeding, and we have in fact employed the modality to stabilize patients with acute hemorrhage from renal AMLs, limitations also exist. First, it should be noted that the histologic diagnosis still remains undiscovered after embolization. Some renal AMLs, although rare, behave aggressively, causing venous tumor thrombus, metastasis and mortality. Endovascular intervention cannot identify the aggressive lesions for more proper management, which could be devastating. Second, the embolization is associated with a higher risk of recurrence than surgery; the secondary treatment rate is 31% after embolization, and is only 1% after surgery [13]. In short, surgical treatment is superior in terms of recurrence and the requirement of secondary treatment. Third, the reported complications associated with embolization include off-target embolization, postinfarction syndrome, necrosis of the tumor and infection, as well as abscesses [12]. Additionally, endovascular intervention is also limited in some situations: allergy to contrast media, multiple or giant aneurysms, a high proportion of fat tissue, which indicates limited benefit from the procedure, and a large ratio of vessels may require retreatment due to vascular rearrangement.

Mirroring the evolution of surgical management for renal cell carcinoma, the surgical intervention for renal AMLs has also shifted from nephrectomy to open partial nephrectomy and then to minimal invasive partial nephrectomy. Although reliance on surgery is challenged by embolization, the role of surgery in the management of bleeding renal AMLs is irreplaceable. The indications for surgery include significant hemorrhage, poor responses to conservative treatments, embolization and suspicion of malignancy [14]. The minimal invasive nephron-sparing surgery has been used to treat both sporadic and tuberous sclerosis-associated AMLs [15–18]. As it has been proposed that the emergency surgical management would likely result in nephrectomy, until now, no studies have focused on this minimal invasive treatment for bleeding renal AMLs in emergency settings.

Here, we described a novel surgical strategy as a possible alternative for treating ruptured renal AMLs. For 3 years, based on advanced surgical expertise and the treatment of a high volume of LPNs in renal cancer, we have been interested in the LPNs management of acute bleeding renal AMLs. As is the case in the artificial pneumoperitoneum, the hyperbaric effect can decrease the oozing from the ruptured tumor, making immediate surgical treatment possible. Based on this premise, we employed the emergency LPNs in the management of bleeding renal AMLs.

Minimizing the interval between rupture and surgery can decrease the possibility of unpredictable re-hemorrhage and avoid the tissue adhesion. Adhesions will occur within 72 h after tissue injury and become more intensive at approximately 10 days to 2 weeks. The adhesive tissue after hemorrhage makes dissection between the kidney and tumor difficult and may inevitably lead to nephrectomy [19]. The advantages of this urgent procedure compared to postembolization surgery include less tissue adhesion, lower financial costs, shorter hospitalizations and the avoidance of the postembolization complications. In our experience, after the initial resuscitation with fluids and blood transfusions, when the patients were hemodynamically stabilized, we immediately performed the surgery and achieved good outcomes.

Compared with conventional LPN for renal tumor, the emergency LPN for ruptured renal AML is relatively difficult. The large perinephric hematoma impedes the mobilization and renorrhaphy, and there is still a risk of bleeding at the beginning of surgery. In the conventional LPN for renal tumors including AMLs, the kidney and tumor mobilizing are prior to the blocking of renal artery. However, for managing the ruptured renal AML, this maneuver might increase oozing before renal artery clamping.

So, we used the following strategies. First, the careful preoperative imaging reading was very important. The surgeon should understand the renal arterial anatomy, the tumor location, the range, and the root well. Although there were hematoma and various degrees of edema around perinephric structures, the anatomic layers were not difficult to be identified in most cases. This might be due to the short intervals between tumor rupture and operation, the fibrosis and adhesion had not happened. We adopted the renal artery priority strategy. Once the renal artery was exposed, unmanageable hemorrhage and possible nephrectomy could be avoided. Thus, we preferred the retroperitoneal approach, as it offered more direct access to the kidney and hilum with limited dissection, which facilitated the most critical part of the operation.

Next, once the renal artery was found and blocked, we dissected the perinephric fat and aspirated the blood clot and tumor to expose the root of the tumor. We do not dissect the tumor completely from surrounding tissues as routine LPN, because it could prolong the warm ischemia time (WIT). After adequate exposure of the tumor root, we resected the tumor from the kidney. The tumor block was pushed away from the kidney by the pneumoperitoneum, allowing space for renorrhaphy.

Then, we applied the continuous, two-layer, unknotted suturing technique [20]. The deep tumor bed was continuously sutured using 3–0 barbed sutures. The renal capsule was reconstructed using 2–0 barbed suture. All sutures were end-loaded with Hem-o-loc clips. The application of the clips decreased the tension and prevented the sutures from lacerating.

Last, after renorrhaphy, we completely removed the tumor tissue and blood clot and placed the drainage tube. As the ultimate goal of LPN was preserving renal function, the key point of this procedure was minimizing the WIT. If prolonged WIT was unavoidable, we moved the clamp off the renal artery after suturing the first layer.

As there was a possibility of malignancy, we also performed intraoperative frozen section as a routine procedure to clarify the pathology. All lesions were subsequently proven to be renal AMLs via histopathology. In this study, we described our experience with emergency LPN for bleeding AMLs. We did not engage in conversion to open surgery or nephrectomy. The mean operative time was 101 min. The mean WIT was 28 min. No intraoperative complications occurred. No patient developed recurrent symptoms, chronic renal insufficiency or dialysis dependence during the follow-up.

Our study specifically focused on the emergency management of bleeding renal AMLs by means of LPN and showed that LPN was safe and efficacious as a minimally invasive method to control bleeding, preserve renal function, and clarify the pathologic diagnosis. Although this emergency utility may be debatable for bleeding renal AMLs, it can be contemplated, as this approach provides a permanent cure for renal AMLs with definite pathological diagnosis. However, the use of this procedure would be limited in several scenarios. First, it is difficult to handle large renal AMLs on the dorsal side of the kidney, as it is difficult to mobilize and expose the renal pedicle. Second, the procedure is not appropriate for multiple lesions and predictable prolonging the warm ischemic time. Third, as this is a challenging technique, the surgeon should possess substantial expertise, particularly with LPN, thereby limiting the widespread use, especially in relatively low-volume medical centers. The methodological limitations of this study include the lack a control group, small sample size, and its retrospective nature with limited follow-up. Ideally, a prospective randomized controlled trial with a large cohort is needed to investigate whether urgent LPN could be used as a routine procedure for selected patients with hemorrhage due to ruptured renal AMLs in emergency settings.

## Conclusion

In this study, we demonstrated that the emergency laparoscopic partial nephrectomy was a safe, minimally invasive procedure for excising the tumor, controlling bleeding, preserving renal function, and clarifying the pathologic diagnosis for emergency patients with ruptured renal AMLs. The procedure can be considered as an effective alternative to renal artery embolization.

## Supplementary information

**Additional file 1: Supplementary Table 1**. Characteristics of all 15 patients.

## Data Availability

The datasets used and/or analyzed during the current study are available from the corresponding author on reasonable request.

## Abbreviations

AML: Angiomyolipoma
TSC: Tuberous sclerosis complex
LPN: Laparoscopic partial nephrectomy
WIT: Warm ischemic time

## References

[1] Flum AS, Hamoui N, Said MA, Yang XJ, Casalino DD, McGuire BB (2016). Update on the diagnosis and Management of Renal Angiomyolipoma. J Urol.

[2] Nelson CP, Sanda MG (2002). Contemporary diagnosis and management of renal angiomyolipoma. J Urol.

[3] Radhakrishnan R, Verma S (2011). Clinically relevant imaging in tuberous sclerosis. J Clin Imaging Sci.

[4] Ljungberg B, Albiges L, Abu-Ghanem Y, Bensalah K, Dabestani S, Montes SFP (2019). European Association of Urology guidelines on renal cell carcinoma: the 2019 update. Eur Urol.

[5] Murray TE, Lee MJ (2018). Are we Overtreating renal Angiomyolipoma: a review of the literature and assessment of contemporary management and follow-up strategies. Cardiovasc Intervent Radiol.

[6] Zhang X, Li HZ, Ma X, Zheng T, Li LC, Ye ZQ (2005). Retroperitoneal laparoscopic nephron-sparing surgery for renal tumors: report of 32 cases. Urology..

[7] Steiner MS, Goldman SM, Fishman EK, Marshall FF. The natural history of renal angiomyolipoma. J Urol. 1993;150:1782–6. 10.1016/s0022-5347(17)35895-0 8230504

[8] Jinzaki M, Silverman SG, Akita H, Nagashima Y, Mikami S, Oya M (2014). Renal angiomyolipoma: a radiological classification and update on recent developments in diagnosis and management. Abdom Imaging.

[9] Lane BR, Aydin H, Danforth TL, Zhou M, Remer EM, Novick AC, et al. Clinical correlates of renal angiomyolipoma subtypes in 209 patients: classic, fat poor, tuberous sclerosis associated and epithelioid. J Urol. 2008;180:836–43. 10.1016/j.juro.2008.05.041 18635231

[10] Pirson Y (2013). Tuberous sclerosis complex-associated kidney angiomyolipoma: from contemplation to action. Nephrol Dial Transplant.

[11] Kothary N, Soulen MC, Clark TWI, Wein AJ, Shlansky-Goldberg RD, Crino PB (2005). Renal angiomyolipoma: long-term results after arterial embolization. J Vasc Interv Radiol.

[12] Jou YC, Chen WP, Huang CL (2009). Urgent Angioembolization with early elective nephron-sparing surgery for spontaneously ruptured renal Angiomyolipoma. J Chinese Med Assoc.

[13] Fernández-Pello S, Hora M, Kuusk T, Tahbaz R, Dabestani S, Abu-Ghanem Y (2020). Management of Sporadic Renal Angiomyolipomas: a systematic review of available evidence to guide recommendations from the European Association of Urology renal cell carcinoma guidelines panel. Eur Urol Oncol.

[14] Wang C, Li X, Peng L, Gou X, Fan J (2018). An update on recent developments in rupture of renal angiomyolipoma. Medicine (Baltimore).

[15] Fazeli-Matin S, Novick AC (1998). Nephron-sparing surgery for renal angiomyolipoma. Urology..

[16] Heidenreich A, Hegele A, Varga Z, von Knobloch R, Hofmann R (2002). Nephron-sparing surgery for renal Angiomyolipoma. Eur Urol.

[17] Msezane L, Chang A, Shikanov S, Deklaj T, Katz MH, Shalhav AL (2010). Laparoscopic nephron-sparing surgery in the management of angiomyolipoma: a single center experience. J Endourol.

[18] Boorjian SA, Frank I, Inman B, Lohse CM, Cheville JC, Leibovich BC (2007). The role of partial nephrectomy for the Management of Sporadic Renal Angiomyolipoma. Urology..

[19] Chang Y-H, Wang L-J, Chuang C-K, Wong Y-C, Wu C-T, Hsieh M-L. The efficacy and outcomes of urgent superselective transcatheter arterial embolization of patients with ruptured renal angiomyolipomas. J Trauma. 2007;62:1487–90. 10.1097/01.ta.0000221051.68550.4a 17563671

[20] Liu X, Ma X, Liu Q, Huang Q, Li X, Wang B (2018). Retroperitoneal laparoscopic nephron sparing surgery for large renal angiomyolipoma: our technique and experience. A case series of 41 patients. Int J Surg.

